# Feathers, folklore, and eco-literacy: Stories ascribe cultural keystone status to avian scavengers in South Asian cities

**DOI:** 10.1093/ornithapp/duae056

**Published:** 2024-10-15

**Authors:** Urvi Gupta, Nishant Kumar

**Affiliations:** Warnell School of Forestry and Natural Resources, University of Georgia, Athens, Georgia, USA; School of Geography and the Environment, University of Oxford, Oxford, UK; Wildlife Institute of India, Chandabani, Dehradun, India; Wildlife Institute of India, Chandabani, Dehradun, India; Dr. B. R. Ambedkar University Delhi, Kashmere Gate, Delhi, India; Edward Grey Institute of Field Ornithology, Department of Biology, University of Oxford, UK; Mansfield College, University of Oxford, Oxford, UK; THINKPAWS Sustainability Research Foundation, Delhi, India

**Keywords:** folk biology, food subsidies, human animal interface, global south, scavenging, urbanization, vulture, शहरीकरण, फ़ूड सब्सिडी, लोक जीवविज्ञान, मानव-पशु संपर्क, वैश्विक दक्षिण, गिद्ध, स्केवेंजिंग

## Abstract

We examined the cultural significance of commensal avian scavenger species—vultures, kites, and crows—and their exploitation of anthropogenic resources and sentiments within Delhi’s urban landscapes. For this, we investigated the intrinsic values attributed to these birds by people, which are indicative of complex, rapidly urbanizing social-ecological systems. Semi-structured interviews revealed folk perceptions intertwined with socio-cultural narratives and traditions, shaped by observations of avian morphology, ecology, and behavior. Birds’ nesting habits, habitats, home ranges, and foraging behaviors affected people’s perceptions, while ecosystem services inspired zoomorphism and anthropomorphism via vernacular-nomenclature (e.g., *chidiya* collectively for songbirds, *giddha* for scavenging raptors). Culturally rooted perceptions, which informed ritual feeding practices and shaped prevalent attitudes toward commensal species, fostered mutual tolerance, and brought people into closer contact with urban biodiversity. Such physical and cultural proximity is a defining characteristic distinguishing tropical urban ecosystems from their Western counterparts. We also uncovered the web of social-technological influences on animal-related folk stories. The urbanization of perceptions in vulture extinction zones revealed shifts in social–ecological relationships with wildlife. It adds cultural dimensions to the currently appreciated keystone status of vultures, vital for their erstwhile coexistence at extremely high densities in South Asia. Urban transformations, technological advancements, and media exposure potentially reshaped human–animal interface, with media misinformation affecting personalized ecologies. Conflicts and health concerns arose from media narratives on garbage-consuming animals. Our findings offer insights to prevent severing of people and nature connections due to urbanization (e.g., technological applications can integrate scientific knowledge with biocultural narratives and folklore), promoting a new-age eco-literacy.

## सारांश

यह शोधपत्र दिल्ली के शहरी वातावरण में गिद्ध, चील और कौवे जैसे पक्षियों के सांस्कृतिक महत्व और उनके द्वारा मानवजनित संसाधनों और धार्मिक भावनाओं के उपयोग पर केंद्रित है। चूँकि शहरीकरण की वजह से हो रहे समाज - पर्यावरण के आपसी जटिलताओं का आकलन आवश्यक है, इस शोध के अंतर्गत हमने लोगों द्वारा इन पक्षियों को दिए गए भावनात्मक मूल्यों का अध्ययन किया। एक विस्तृत प्रश्नावली पर आधारित साक्षात्कारों से हमें ज्ञात हुआ कि इन पक्षियों से जुड़ी लोक धारणाएं सांस्कृतिक कथाओं और परंपराओं से प्रभावित थीं। हमने पाया कि क्षेत्रीय नाम और लोकोक्तियाँ पक्षियों की शारीरिक रचना, पारिस्थितिकी और व्यवहार के अवलोकनों से आकार लेती हैं। पक्षियों की घोंसले बनाने की आदतें, आवास, क्षेत्र की सीमाएं और भोजन लोगों की धारणाओं को प्रभावित करते हैं, जबकि इनके इकोसिस्टम सर्विस स्थानीय नामकरण के माध्यम से उपनामों और लोकोक्तियों को प्रेरित करते हैं, जैसे कि, ‘चिड़िया’ छोटे एवं सुरीले, और ‘गिद्ध’ मृत शरीर खाने वाले पक्षियों के लिए। सांस्कृतिक धारणाएं पशु - पक्षियों को भोजन देने की प्रथाओं के प्रचलन का आधार हैं, जो इंसान और सहजीवीयों के सह-अस्तित्व को आकार देती हैं। इस आपसी तालमेल से पनपी सहिष्णुता लोगों को शहरी जैव-विविधता के करीब लाती हैं। ऐसा भौतिक और सांस्कृतिक मेल विकासशील देशों के शहरी इकोसिस्टम (पारिस्थितिकी तंत्र) को पश्चिमी शहरों से अलग करता है। हमने पशु-संबंधित लोक कथाओं पर सामाजिक-तकनीकी प्रभावों का भी पता लगाया। गिद्ध विलुप्ति क्षेत्रों में धारणाओं के शहरीकरण ने वन्यजीवों के साथ मनुष्य-पर्यावरण संबंधों में बदलाव का खुलासा किया। यह स्थिति गिद्धों की वर्तमान में सराही गई कीस्टोन स्थिति में सांस्कृतिक आयाम जोड़ती है। कालांतर में ये सांस्कृतिक आयाम दक्षिण एशिया में गिद्धों के अत्यधिक घनत्व और उनके पूर्व सह-अस्तित्व के लिए महत्वपूर्ण थे। शहरी परिवर्तन, तकनीकी प्रगति और मीडिया एक्सपोजर ने मानव-पशु इंटरफेस को संभावित रूप से पुनः आकार दिया, जिसके अंतर्गत मीडिया के प्रभाव और गलत जानकारीयों ने व्यक्तिगत इकोलॉजी को प्रभावित किया। मृत पशु और अपशिष्ट खाने वाले जानवरों पर मीडिया कथाओं के प्रचलन की वजह से स्वास्थ्य संबंधी चिंताएं उत्पन्न हुई हैं। इस शोध के निष्कर्षों में हमने शहरीकरण के कारण लोगों और प्रकृति के संबंध-विच्छेदन को रोकने के उपायों पर प्रकाश डाला हैं। उदाहरणार्थ, तकनीकी अनुप्रयोग (सोशल मीडिया) के तहत जानवरों के प्रति नवीन वैज्ञानिक दृश्टिकोण को जैव-सांस्कृतिक कथाओं और लोक-कथाओं के साथ जोड़ एक नए युग की पारिस्थिति की साक्षरता को बढ़ावा दिया जा सकता है।

## INTRODUCTION

Human population growth and urbanization are significant threats to biocultural heritage today (Turner et al. 2004). Developing countries are expected to account for 98% of this growth, leading to drastic transformations in their social–ecological landscapes (Anonymous 2016). Urbanization is often linked to homogenization of biota and human–wildlife interactions (Alberti 2008), severing urban immigrants from their ancestral biocultural heritage (Soulsbury and White 2016). Such physical and cultural changes affect people’s attitudes toward non-human species and shape their conservation priorities (Miller 2005, Soga and Gaston 2020). Interactions between humans and nature have a significant impact on biodiversity at the urban–rural interface, particularly in Asia and Africa. These regions are projected to house 75% of the global urban population by 2050, primarily through the development of megacities (Anonymous 2016).

Tropical cities exhibit distinct ecological and biocultural profiles compared to their temperate counterparts. Such developing centers reflect the interplay between infrastructural aspects of urbanization and the intangible socio-cultural heritage (Kumar et al. 2018a, Nagendra 2018). This eastern–western disparity is evident in the responses to biodiversity among residents of towns and cities based on their socio-cultural and economic backgrounds. A factor contributing to these pattern disparities is the prevalent “central core-to-periphery” design found in Western cities that were purposefully planned. In contrast, cities located in the Global South are typically characterized by heterogeneous development (Philol 1995). Such differences underscore the importance of regional contexts when examining urban ecological patterns and cultural attitudes toward nature. However, the prevailing orientations in urban ecology, particularly in understudied tropical cities, tend to overemphasize biophysical perspectives, thereby neglecting the intricate social–ecological characteristics of urban ecosystems (Myers 2021).

Urbanization is often depicted as a physical, abiotic force, generating perennially disturbed systems. But recent scholarship has recognized urban ecosystems as social-ecological–technological systems (SETS), a definition that encompasses more integrated and multifaceted views of interactions among natural, social, and technological components (Grimm et al. 2017). The exploration of biophysical-biocultural connections and their role in shaping novel SETS is important (Collier 2015), considering that cities occupying just 3% of the Earth’s land area are home to > 60% of the global human population (UNO 2018).

The influence of urbanization on ecology, particularly through the lens of regional social traditions and practices, has not been sufficiently explored across scales. This oversight necessitates a comprehensive examination of the complex interplay among urbanization, social traditions, and urban ecology (Iaccarino 2003). While recent research has primarily focused on characterizing human–animal relationships by investigating the behavioral mechanisms that facilitate coexistence through mutual adaptations (Bermúdez-Cuamatzin et al. 2009, Lowry et al. 2013, Møller and Ibáñez-álamo 2012, Newsome et al. 2015), very few studies have examined the complex reciprocation between urban animals and human socio-economic and cultural aspects in tropical megacities, which may influence their resilience and coexistence with humans (Bhatia et al. 2021, Kumar et al. 2018b, Myers 2021, Nagendra 2018).

Previous studies have highlighted the adverse effects of anthropogenic activities and infrastructure on species richness (Alberti 2008). Yet, certain species have thrived in human-dominated landscapes (Marzluff 2017). This synurbanism requires coordinated efforts—termed co-cultures—from both human and non-humans to develop behavioral and cultural strategies that support coexistence (Francis and Chadwick 2012; Sueur and Huffman 2024). In tropical regions, where such species have coexisted with humans for centuries, non-humans have become embedded in local folklore and are integral to social customs and rituals. These cultural perceptions influence human–wildlife interactions and consequently, public will—a critical factor for biodiversity conservation (Hosey and Melfi 2014, Hulme-Beaman et al. 2016, Kumar et al. 2019c, Oro et al. 2013, Soulsbury and White 2016).

The dynamics of coexistence and escalating conflicts over intensified human–animal interactions create unique tensions at the backdrop of socio-cultural and infrastructure changes in tropics, particularly in South Asia (Dhee et al. 2019, Ghosal and Kjosavik 2015). For example, in Delhi, attitudes toward *Milvus migrans* (Black Kite) have shaped accommodative human responses to their offspring defense behaviors exhibited as attacks on people’s heads. Such attacks are often seen as “parental obligations” in the region. Beliefs linking the well-being of humans and animals play a crucial role in mitigating conflicts, beyond cultural boundaries (see Kumar et al. 2019a, 2019b, 2019c). However, ecologists are yet to fully grasp the homogenizing effects of urban-cultural convergence, particularly among younger generations less inclined toward traditional practices like ritualized feeding and tolerance (Kumar et al. 2020a). The predictable nature of anthropogenic resources, combined with human beliefs and practices that promote mutual acceptance, leads to habitat mosaics with varying degrees of suitability for diverse synurbic species (Barua 2010, Bhattacharjee et al. 2018, Hosey and Melfi 2014, Kumar et al. 2019a, Møller and Ibáñez-álamo 2012).

In this study, we delved into the complex relationship between human cultural practices and urbanization, examining the influence of co-cultures on wildlife coexistence within urban settings. Our primary focus was on the cultural characterization of urban commensal birds by people, achieved through dialogs with thousands of casual observers during our fieldwork on the nesting ecology of *M. migrans* in Delhi, India (Kumar et al. 2014, 2018a). This approach is significant for several reasons. First, cultural tolerance toward certain animal species can result in habitat over-selection by specific behavioral phenotypes, where proximity to humans offers foraging advantages in the form of food-subsidies (Bhatia et al. 2021). Such mutual behavioral adaptations, often linked to *biophilia* (Kellert and Wilson 1993), result in demographic consequences that shape the cultural-behavioral dynamics at the human–animal interface across spatial and temporal dimensions (Cox et al. 2018, Lepczyk et al. 2004, Robb et al. 2008). Second, the culture-mediated presence and abundance of common, commensal species that become integrated into urban landscapes, influence regional human connections with nature (Cox et al. 2018, Fuller et al. 2008). Finally, analyzing human socio-cultural perceptions of urban species can provide insights into potential variations in their behavioral and demographic traits (Kumar et al. 2019a, 2019b), which may have implications for their long-term management within urban ecosystems.

Within the framework of the mutual human–animal adaptations, our paper articulates the relationships between people, birds, and places, illustrated through biocultural narratives. Through semi-structured interviews, we explore human attitudes toward avian scavengers (target species: vultures, kites, and crows) in the tropical megacity of Delhi, India (Galushin 1971). The decline in the vulture (*Gyps* spp.) populations across the Indian Subcontinent has provided an opportunity for quasi-experimental research (Prakash et al. 2003). This led us to investigate the socio-cultural shifts associated with the invasion and stabilization of animal species undergoing ecological succession and illuminated the distinctions between the ecological and cultural keystone statuses of vultures. Our ethnography focused on the consequences of the detritus removal by obligate/facultative scavengers in urban ecosystems (Soga et al. 2020, Soga and Gaston 2016). Our decade-long monitoring of kite populations in Delhi—both resident and migratory—has provided insights into how a tropical megacity accommodates common scavenging birds within backyards, facilitating direct observation and interaction by residents. Moreover, we investigate how residents’ perceptions of *Corvus splendens* (House Crow) are shaped by the crows’ nuanced behavioral repertoire, reflecting urban adaptations (Marzluff and Neatherlin 2006). Based on the dialogs with thousands of casual observers during our long-term fieldwork, we used ethnography to test the hypothesis that morphological attributes and regional behavioral repertoires, captured and rewired by folklores, play a significant role in the persistence of select non-human species in human-dominated landscapes. We also address the extinction of experience (Pyle 1993, Soga et al. 2020) concerning common backyard fauna and argue for the necessity of inter/transdisciplinary approaches in understanding and managing these dynamics. The primary objective of our research was to delve into relationships between the city’s inhabitants—who come from different parts of the Indian subcontinent—and the commensal avian species (Bhagat and Mohanty 2009).

## METHODS

### Study Area

Delhi, India, seated within a network of rapidly expanding megacities, is home to over 30 million people and spans an area of 1,500 km^2^ (Census India 2011, UNO 2018). The national capital attracts people from all over the country for livelihood. The city’s layout is polycentric and diverse, featuring a variety of urban configurations that defy a linear urban-rural gradient, emblematic of South Asian urbanism (Shahmoradi 2013). Delhi’s climate is semi-arid, with an average annual rainfall of 640 mm that primarily occurs during the monsoon season in July and August. The city experiences a wide temperature range, with average minimum temperatures of 8.2°C in winter and average maximum temperatures of 39.6°C in summer (IMD 2022). The regional vegetation is classified as “Northern Tropical Thorn Forest” (Champion and Seth 1968).

### Urban Waste Metabolism and Opportunistic Scavengers

**FIGURE 1.**
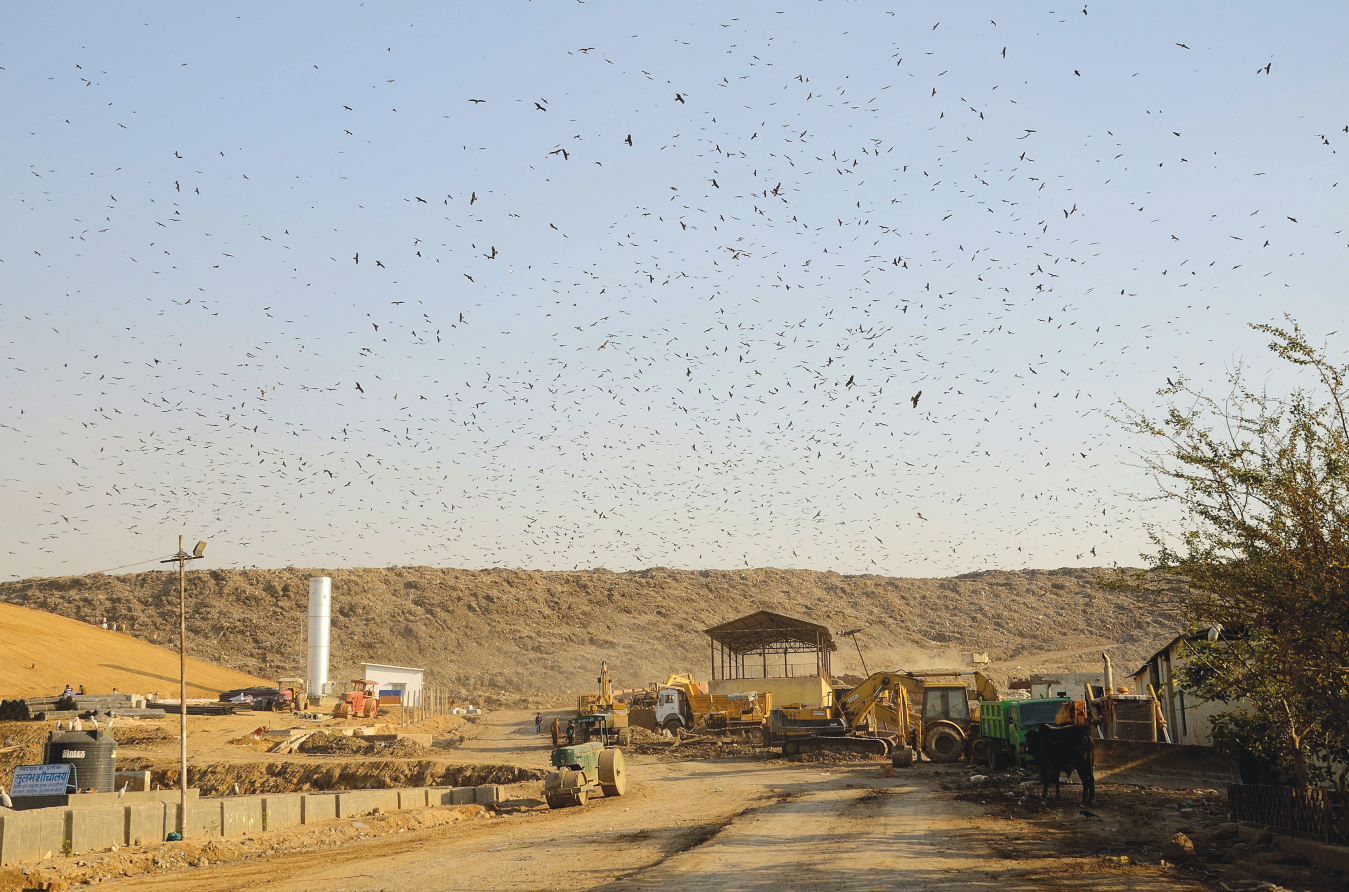
Massive flocks of *Milvus migrans* (Black Kite) are a common sight at landfills in tropical cities across South Asia. This is a striking image of over 10,000 kites congregating over and around the Ghazipur landfill in Delhi, India, which covers an area of 70 acres; they mainly feed on poultry processing waste (photo credit: Fabrizio Sergio).

Delhi displays 3 critical aspects that likely influence the interactions between humans and avian scavengers. First, inadequate solid waste disposal systems have led to the emergence of informal livelihoods, which are central to waste processing and contribute to the persistence of roadside garbage piles (see Kumar et al. 2019c for details). These piles vary in size and content, fostering close interactions among the urban poor, various facultative avian scavengers (such as kites, crows, egrets, and pigeons), and opportunistic mammals like stray dogs, rats, and cattle (Figure 1). The presence of this detritus and associated non-human organisms imparts a distinctive visual and olfactory ambiance to tropical urban settings like Delhi (Gupta and Gupta 2017), epitomizing the sensory aspects of tropical urbanization (Doron 2021). Second, increasing meat consumption in and around Delhi is creating subsystems of animal waste. These changes are leading to increasing volumes of unsegregated waste and animal byproducts, and a scarcity of suitable waste disposal sites. Third, conflicts have arisen among various civic authorities tasked with waste management, leading to an increase in negative perception and low tolerance about certain animal scavengers (Kumar et al. 2019c).

### Human and Urban Parameters of Facultative Avian Scavenging

This study is part of a long-term research initiative launched in 2012 (Kumar 2013), aimed at unraveling the ecology and ethno-zoology of urban scavengers in Delhi. Our approach has been methodical, involving systematic breeding kite monitoring surveys across 32 sampling plots, each ~1 km² in size, selected through a stratified-random design. These plots represent a diverse array of urban environments, ranging from semi-natural areas to densely populated regions, reflecting the city’s varied socio-cultural fabric. The design included all 3 sanitary landfills (for details, see Kumar et al. 2019a). This has facilitated interactions with an average of 27.03 ± 2.31 new observers daily within each sampling unit, providing a rich source of qualitative data on human–avian coexistence in the context of urban waste management.

### Data Collection Approach

**FIGURE 2.**
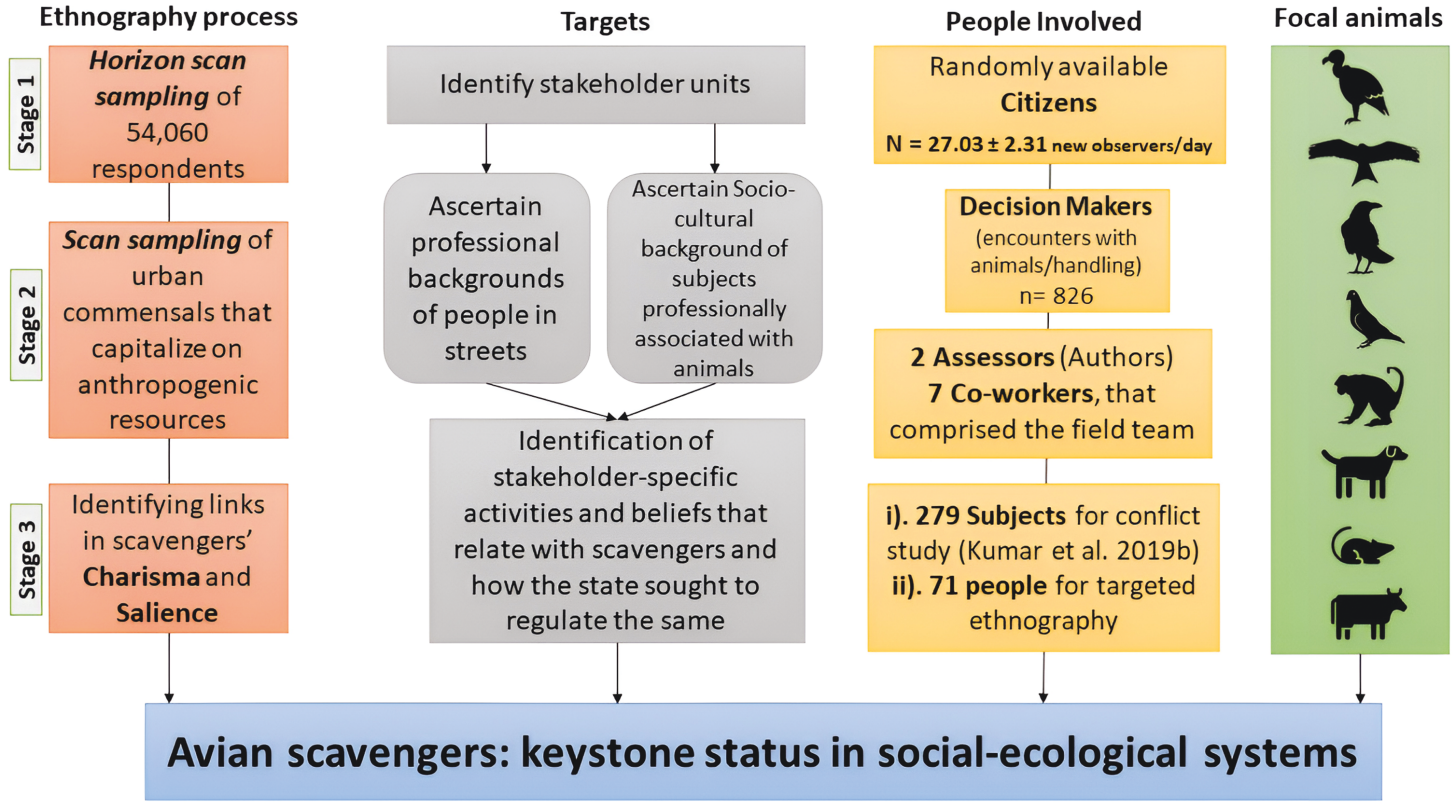
Methodological stages (adopted and modified from Esmail et al. 2020) illustrating how people involved in opportunistic surveys and focused ethnography participated at each stage of assessment for human–avian scavengers’ co-cultures.

We adopted a Delphi-like ethnographic method (Mukherjee et al. 2015), inspired by the horizon scanning technique described in Esmail et al. (2020). Horizon scanning is increasingly recognized as a valuable tool in making informed decisions by providing a structured way to anticipate future developments and their implications. The technique is relevant in fields such as environmental management, public health, and technology policy, where rapid changes can have significant consequences. We incorporated this approach for evaluating collective judgment (Figure 2). It allowed us to deepen our understanding of the factors driving coexistence with avian scavengers. The ethnographic process unfolded in 3 sequential stages:

#### Stage 1: Gathering preliminary data

We capitalized on spontaneous gatherings of observers from diverse socio-cultural backgrounds, who were drawn to our field activities (see Kumar et al. 2014, Supplementary Material S1). This stage established a link between the ecology of our focal species—kites, crows, and previously common vultures—and the influence of urban infrastructure, human activities, and religio-cultural beliefs on birds’ access to anthropogenic food sources (Kumar et al. 2019a). Our interactions with these people laid a foundational understanding of local knowledge and perceptions, which we enriched through iterative discussions, identifying various degrees of engagement and associations between stakeholders and the avian species under study.

#### Stage 2: Thematic organization and environmental assessment

As we monitored the evolving ecological dynamics of avian commensals over several years, we engaged with new observers at our consistent monitoring locations, assessing the persistence of perceptions identified in Stage 1. We organized reactions and responses to our field work and conversations thematically, segregating how bird/environmental factors mediated avian scavengers’ charisma and salience before each iteration (Figure 2). This thematic organization helped address the questions pertaining to relationships between respondents’ socio-cultural disposition toward avian scavengers and their professional/social backgrounds. Our categorization exercise provided insights into the factors influencing perceptions and interactions with avian scavengers and other commensals. Concurrently, we assessed the environmental variables in the vicinity to understand how varying availability and accessibility to anthropogenic resources shaped the nature of interactions—prosocial or agonistic—between people and animals (avian scavengers, dogs, other commensals, and livestock) in the study area (Figure 2).

#### Stage 3: Semi-structured key informant interviews for social-ecological exploration

The presence of onlookers during kite monitoring fieldwork facilitated the acquisition of a substantial mean sample size of 54,060 respondents (standard error: 4,620). This was achieved through ~ 250 field visits conducted annually from 2012 to 2020. After the categorization of potential respondents into distinct stakeholder groups within our sampling units, we implemented a random sampling and a snowball sampling technique to capture a wide array of viewpoints (Newing 2010). From a total pool of 826 decision makers (Figure 2), the categorization (detailed within Supplementary Material 2), encompassed: (1) academic experts (5.7%): researchers and academics specializing in ecology and conservation, and often pursued birding; (2) non-governmental organizations (NGOs) (5.5%): members from organizations like the World Wide Fund for Nature and the Wildlife Trust of India, committed to wildlife preservation, the Jain Bird Hospital and Rescue Center, and other rescue and rehabilitation centers; (3) ritual feed-sellers (2.4%): individuals engaged in selling grains, fruits, and meat for ritualistic feeding of birds; (4) government professionals (9.3%): employees across various government sectors, including municipalities and animal health services, with involvement in animal husbandry and management of wild or feral animals in shared urban spaces or zoological parks; (5) religious organizations (2.1%): had priests and Muslim clerics from diverse religious institutions; and (6) environmental and occupational stakeholders (75%): individuals in environments or occupations where food supplementation or waste availability attracts opportunistic scavengers, including butchers, market cleaners, rag pickers, people feeding animals, and municipality contract workers. A few of our respondents overlapped these categories. We also divided respondents by age: those 35 years and older and those between 18 and 35 because an extinction of vultures occurred in the 1990s. This age-based segmentation aimed to shed light on the extinction of experience and its repercussions on social-ecological systems (Soga et al. 2020, Soga and Gaston 2016).

Based on the first and second stages of our ethnographic investigation, we deviated from the traditional reporting of respondent proportions, which is typically based on quantitative aspects of interviews, and frequently utilized in the natural and social sciences. Instead, at stage three, we opted for an alternative approach, where we explored why the conventional reporting of proportions might not be the most effective means of capturing the dynamics of human perception of animals. We employed semi-structured interviews to gain insights into how popular perceptions often originate from a limited number of impactful stories that gain widespread dissemination. Furthermore, we anticipated that specific species and local contexts (involving the interactions between birds and human environments) influence how these stories are further elaborated upon. Our study prioritized qualitative analysis, acknowledging that popular perceptions about commensals were most likely not influenced by numerical representations. We conducted semi-structured key informant interviews (*n* = 71 in 2017) to explore the relationships between diverse avian scavengers and the focal groups (Supplementary Material 2). This approach allowed us to map the geography of human–scavenger relationships, urban opportunism among commensals and the co-cultures that underpin social–ecological systems in shared landscapes (Kumar et al. 2019a, b, c; Sueur and Huffman 2024).

The effectiveness of horizon scanning, as a retrospective evaluation (Sutherland et al. 2019), provided insights into the behavioral patterns of avian scavengers and the dynamics of real-time human–avian interactions across various spatial and temporal contexts. These data shed light on the dynamics of real-time human–avian interactions, involving individuals from various communities, religious affiliations, and professions. We used thematic analyses based on the interview transcripts. These transcripts were anonymized to ensure confidentiality in keeping with human subjects’ ethics permits held at the primary affiliate institutions. This process involved coding responses to identify patterns and themes within the data, which were then interpreted for links in people-places and birds. Qualitative data gathered through semi-structured interviews elicited comprehensive and insightful responses from the participants. Interviews were either audio-recorded with the consent of the participants to ensure accuracy and reliability or summarized on paper as soon as the interview was finished. The recorded data were then transcribed verbatim to facilitate a thorough analysis. Qualitative data were analyzed using a systematic approach. The transcribed data were read multiple times to gain an understanding of the participants’ perspectives. Then, the data were coded into themes and sub-themes, a process that involved identifying and categorizing patterns in the data (Esmail et al. 2020, Mukherjee et al. 2015). Interviews from stages 1 to 3 were conducted by the team in the most appropriate language, based on the respondents’ comfort in *Hindi*, *Bhojpuri*, *Maithili*, *Bengali*, *Assamese*, and English. It ensured effective communication. For an in-depth look at the semi-structured interview methodology, refer to Supplementary Material 1 and 2.

## RESULTS

The relationships between humans and avian scavengers in Delhi’s urban environment were shaped by several factors, including direct and indirect experiences and a collective responsiveness to local biodiversity. Our research interviews revealed a diverse vocabulary used by residents to describe avian species, summarized in Table 1 and Supplementary Material 2 and 3. Thematic categorization (stages 1 and 2) from our ethnographic exercise revealed that this vocabulary was influenced by the birds’ vocalizations, morphological characteristics, and the socio-geographical context of the respondents. Factors such as immigration status, type of residential area, and varied urban experiences contributed to the terminology used. Similar to how taxonomists classify species based on multiple morphological traits, the local populace’s terminologies, perceptions, and practices regarding avian scavengers were intertwined with traditional folklore. This species-specific folk biology, such as the higher synurbization of crows, is captured in the prevalent notion that a “crow’s caw signals the arrival of a guest”; “crow’s arrival signal God of Death, Yam”; “crows bite people who lie.” Such statements are urban legends, and events are mere correlations: the synurbization of crows ensures that some bird will inevitably caw at the door, concurrent to frequent arrival of guests in the familial setup of India. This illustrates how the opportunistic ecology of a species becomes entwined with cultural practices in India, which celebrates arrival of guests. Additionally, the belief that “ancestors take the form of crows or dogs,” animals that are most likely to consume food offerings during the fortnight (September–October) of respecting the dead (*pitra paksha*), highlights how cultural and ecological interactions give rise to such notions. The emergence of co-cultures involving avian scavengers was likely influenced by reciprocal advantages and disadvantages associated with proximity to human settlements, particularly among urban poor, immigrant communities, and individuals engaged in livestock rearing. Perceptions that mediated religious practices associated with avian scavengers were rewired by folklore (summarized in Table 1 and Supplementary Material 2 and 3).

**TABLE 1.** Diversity of cultural associations and dominant perceptions people in Delhi have for the three target groups of avian scavengers.

Species	Abundance and experience	Behavior and perceptions	Cultural association	Stories (a form of media)
*Corvus splendens* (House Crow)	Abundant, highest proximity, backyard interaction	Intelligent, timid, shy, bold, communal birds that mob in flocks, congregates for a dead crow, caws on nest intrusion; nests near humans	Highest for Hindus, offered different foods by Hindus and Muslims such as bread, rice pudding, grains and meat	Maximum; folks- legends, fables, myths depicting intelligence across all age groups and cultures (e.g., Crow and the pitcher)
*Milvus migrans* (Black Kite)	Abundant, moderate proximity, interaction from a distance, snatched food from hands	Attacks, snatches eyes, elegant flight, snatches food from hands, nests far from humans in jungles and eats meat, rat, roadkill	Mythological for Hindus though not as popular as crows; high association with Muslims in Delhi, followed by Rajputs and Kashmiri *Pandits*	Mostly of conflict and attack for food or nest defense (eye snatchers)
*Gyps* spp (Vultures)	Critically endangered since 1990s, extinction of experience in city for almost two decades	Does not attack people or animals, congregates over dead, nests far off in jungles and mountains	High for Hindus and Parsis; fed by Muslims; now not spoken about since their decline	Mostly mythical/cultural- Jatayu (Hindus, age 50 and above), with keen eyesight

Stages 1 and 2 of ethnography revealed that folk biology was richest among the urban poor within immigrant communities and/or those who rear livestock. Among the stakeholders, individuals residing in the city and professionals in their respective fields placed greater emphasis on scientific viewpoints (Figure 2). Our thematic analysis from stage 2 identified a set of factors that likely mediated the co-cultures in social–ecological systems influenced by human–avian scavenger interactions. These 5 thematic factors listed below can be projected in 3 primary categories: ecological, social, and cultural.

### Five Key Thematic Factors

#### Human social factors

The socio-economic conditions of urban residents, particularly those from lower and middle-class backgrounds, shaped their interactions with the city’s infrastructure and biological diversity. For the urban poor, such as women residing in slums, socio-economic status often confined them to their immediate neighborhoods, limiting their exposure to the city’s broader offerings and autonomy to connect with nature. In contrast, their male counterparts had opportunities to traverse multiple sections of the city due to different occupational roles. This dichotomy highlighted how economic constraints and cultural norms could restrict access to resources and experiences. The degree of belongingness and engagement with the city’s infrastructure and natural environment was thus mediated by one’s residential location—whether in the city’s heart or its periphery—and the autonomy afforded by their economic, religious, gender, and cultural circumstances.

#### Human psychological factors

The responses provided by urban immigrants highlighted stress due to work and other responsibilities. It led to reduced autonomy in their lives regarding expressions for nature and biodiversity that we registered in ethnography. Their responses encompassed a spectrum of emotions, ranging from stress to contentment that affected their sense of belonging within cities. Hence, personal histories, the duration of residency in one or more urban areas, educational achievements, and professional roles collectively influenced individuals’ psychological responses to their urban lives and the living.

#### Opportunities for direct experience with birds

Direct experiences between people and avian scavengers were influenced by the spatial distribution of anthropogenic resources, individual- or population-level response to infrastructure along the urban gradient, and their choice of proximity to humans. These interactions were often opportunistic: birds responded to the availability of food subsidies by humans. The degree of habituation to human presence varied among species, affecting the type and mechanism of ecosystem services attributed to each target species we studied. For example, individuals (especially those above age 40) recalled vultures due to their consistent association with carcass sites, as well as their ecological function and a comparatively lower mutual tolerance for proximity. In contrast, accounts of kites and crows encompassed instances of a higher level of synurbization and consequently, tolerance for human proximity. This was reflected in relatively richer cultural contexts of these birds in local folklore, in addition to their perceived roles in scavenging. Species-specific habituation not only influenced direct interactions with humans but also established a foundation for cultural narratives.

#### Cultural narratives shaping indirect associations

Our ethnographic study revealed that indirect associations shaped public perceptions of the avian species in focus. Respondents recounted cultural or urban-legend narratives, exemplifying their enduring belief in *M. migrans* as eye snatchers, despite the absence of direct experiences of attacks. It highlighted the profound influence of folklore and mythology. Their anecdotes revealed that contemporary media and information technology have played a pivotal role in translating regional language fables and disseminating stories pertaining to crows, kites, and vultures. For instance, the advent of televised media at the turn of the century and social media platforms post-2015 have facilitated the portrayal of avian scavengers in nature documentaries as well as prominent Indian mythologies and fables, such as the *Ramayana, Mahabharata*, *The Jungle Book* and *Panchtantra*. These mediums have served as conduits for narratives and anecdotes, shaping biocultural and mythological perceptions.

This study’s ethnographic coverage across diverse socio-economic groups highlighted that caste and class significantly influenced perceptions of the changing urban ecology concerning avian scavengers. The economically disadvantaged urban populace, which typically comprises individuals from lower social castes or economic class, has limited access to media. These individuals recalled aspects of folklore that were distinct from those held by their relatively wealthier counterparts with better access to media. For example, respondents from the urban poor believed that birds of prey, such as vultures and kites, nested in high mountains and flew above the clouds (Table 1). Given that kites typically nest in proximity to residential blocks in Delhi, unlike the native regions of these urban immigrant poor, these responses reflect the influence of regional ecologies on localized folklore and traditional knowledge. While respondents generally appreciated crow’s intelligence, people linked anecdotes, such as the Crow and the pitcher (or Thirsty Crow) story that attribute higher cognition to crows, which have been mediatized through story books such *Aesop*’s fables or *Panchtantra* etc. and brought to screen via animations.

#### Innate responses to biodiversity and social–ecological dynamics

Our research indicated that the innate human affinity for biodiversity, often referred to as *biophilia*, elicited a range of responses from individuals within the urban landscape. This biophilic tendency led to varying degrees of care and attention toward different avian species, influencing the integration of biodiversity into human lives with diverse impacts—some tangible and others less so. Residents reported assisting certain bird species, including the target taxa for this research, citing a moral duty to aid those perceived as weaker amidst “limited food availability.” This sense of socio-religious morality emerged as a key driver in the tolerance of avian scavengers in close proximity, as people recognized the displacement of wildlife due to urban development. For a comprehensive understanding of these dynamics, refer to Table 1 and Supplementary Material 3.

### Avian Scavengers in Social-Ecological Systems

People’s perceptions revealed that ecosystem services provided by specific avian taxa influenced their vernacular nomenclature. Small songbirds, despite having distinct ecological roles were referred to as “*chidiya*.” Similarly, raptors, despite noticeable differences in size and appearance, were often referred to as “*chhota giddha* (small vulture)” or “*bada giddha* (large vulture),” although it means “vulture” in Hindi, indicating an extinction of experience with various raptor species. Out of 71 people interviewed in stage 3, 19 respondents—who knew only Hindi—intermixed kites and vultures’ vernacular names. They used *giddha*, or another generic term *baaz*. *Baaz* is used colloquially for birds of prey that frequently hunt live prey. Mistaken folk nomenclature could be an aspect of “extinction of experience” or morphological and scavenging service similarities (details in Supplementary Material 3). Without any prompting by the authors, 20% of non-birders noted differences between *C. splendens* and *C. macrorhynchos* (Large-billed Crow), discussing beak size and shape. While crows were recognized for their plumage and long, slender, and straight beak, kite’s beak was not mentioned, except by respondents who were experts in wildlife/animal biology or zoo professionals.

**FIGURE 3.**
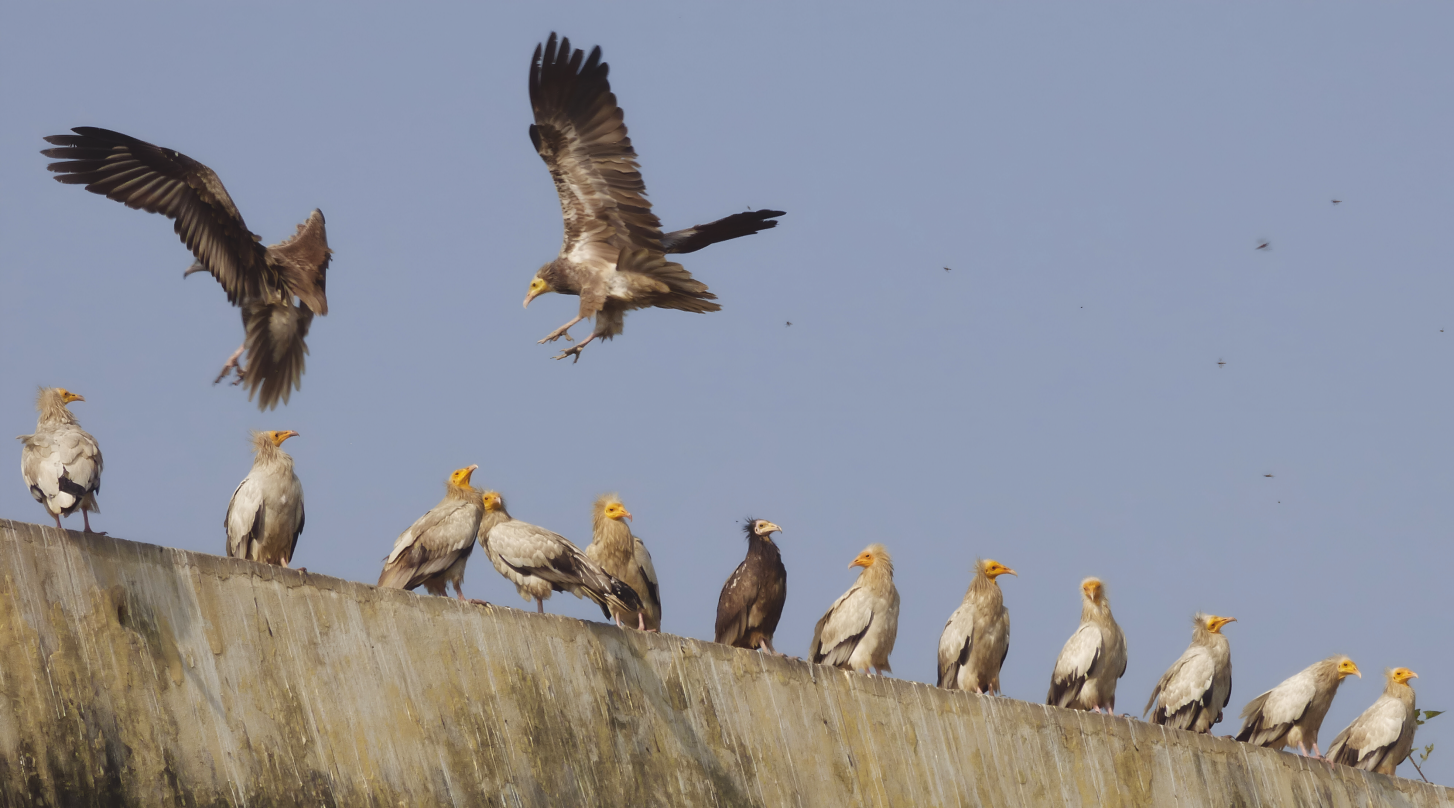
Image of *Neophron percnopterus* (Egyptian Vulture) in juvenile (dark) and adult (light) plumages. The dark juvenile plumage resembles the plumage of black kites, leading to the vernacular naming of kites as “*kaali* (Black) *cheel*” and vultures as “*safed* (White) *cheel*.” The image also underscores the cultural significance of these birds, with the kite (*cheel*) referred to as the “*nani*” or maternal grandmother of all birds, and the vulture (*safed cheel*) considered the “*Cheel ki naani*” or grandmother of kites.

Respondents, especially those below 35 years old, were unsure about the appearance of vultures, vaguely describing them as large birds with an enormous wingspan. Most respondents mentioned the difference in size, identifying vultures as several times larger than kites and having long necks. This explains why they discounted the *Neophron percnopterus* (Egyptian Vulture), which has a markedly different plumage, neck and head compared to other vulture species, and called it *safed cheel* (a white kite) and *cheel ki nani* (kite’s grandmother). Such adnouns likely stemmed from the assumption that white feathers signify old age (Figure 3). This perception might have been influenced by the plumage similarity between juvenile and subadult morphs of *N. percnopterus* and kites. The human familial structure, an important trait of South Asian societies, extended to avian scavengers. It was apparent in Muslim respondents’ use of endearing terms such as *pakshiyon ki Naani* (translating to the maternal grandmother of all birds), referring to kites. This attribution could be influenced by the kite’s agile and graceful flight, which evokes a sense of sophistication and experience. This observation underscores the deep cultural connections between humans and nature in these societies.

Interviews with individuals who engaged in feeding multiple species revealed how their religio-cultural upbringing influenced their patronizing attitudes. Notably, respondents shared expressions that conveyed a sense of familiarity and recognition between themselves and the birds (e.g., “these birds recognize me and are prepared to be fed”). However, individuals denied the prevalence of ritual feeding in smaller towns and villages. While kites and crows were occasionally referred to as bad omens, they were not generally feared for their size. In contrast, vultures, with their bald heads and necks, large size, and association with death, were described by most respondents as birds that are linked with bad omens. However, respondents did not express disdain, and rather lamented their loss.

### Avian Scavengers and the Extinction of Experience

Ethnographic research demonstrated relationships between the region of origin and the level of folk-systematic knowledge possessed by individuals. Notably, younger wildlife researchers and animal welfare professionals who were natives of Delhi exhibited a relatively diminished familiarity with traditional folklore. Aside from the fact that landfills were previously inhabited by vultures before their local extinction, respondents had a restricted comprehension of patterns in the population of crows and kites. This was based on their visual memory of having observed vultures in action prior to their regional extinction(s) in the 1990s. Consequently, only individuals aged 35 or older who had witnessed vultures within their respective native regions provided accounts, elucidating the appearance and behavior of those avian scavengers. This age group also recalled kites snatching food from hands while eating in the open, a practice not common nowadays.

During the discussions, respondents deliberated upon the environmental and ecological ramifications of vulture loss that are a keystone species. Anecdotal accounts pertaining to direct encounters with vultures primarily revolved around carcass dumping sites located in villages or localities that utilized vulture scavenging services. In the absence of vultures, livestock carcasses were either dumped in open water bodies, buried underground with salt, or discarded in open areas, depending on the financial standing of the owner. The lack of vulture services limits areas in cities where the urban poor can practice animal husbandry. Several respondents frequently expressed apprehensions regarding the escalation of conflicts involving dogs that opportunistically consumed discarded animal remains. Occasionally, people linked the local extinction of vultures with the proliferation of stray dogs. This phenomenon is known as competitive release and has potentially augmented the population density of stray dogs and rodents, which could affect growing concerns about human-canine conflicts and the transmission of zoonotic diseases. Experts who were professionally engaged in routine interactions while working for municipalities, zoological parks, and NGOs dedicated to animal welfare and conservation shared firsthand experiences (see Supplementary Material 1 and 2).

Delegates representing dairy unit establishments situated near *Ghazipur* landfill, which is currently slated for relocation to the northwestern region of Delhi, engaged in a discourse regarding the impact of vulture depletion on the existing and emerging conflicts associated with the disposal of animal carcasses and waste. This discussion highlighted the implications for the viability of sustaining dairy settlements within urban environments such as Delhi (summarized in Supplementary Material 1 and 2). In their responses, people expressed concerns about human degrading natural habitats, which drove their tolerance. The interviews informed that despite variations in perceptions about avian scavengers due to varying cultural and religious beliefs, all stakeholders shared explicit sympathy for non-human life forms. These accounts derived from our ethnographic investigations emphasize the extensive implications of urbanization and heterogeneous development on human behavior toward their non-human co-inhabitants.

Our research revealed that “extinction of experience,” a concept that encapsulates the loss of connections between humans and nature, is not solely attributable to the local extinction of specific species such as vultures. It also stems from the diminished opportunities for interaction, even when species coexist within the same ecosystem. For instance, experiences for female (and young) respondents from informal settlements, whose lives are restricted to their shanties adjacent to garbage accumulations, and whose professional aspirations are confined to the local region, along with vultures and kites, were indirect. Their perceptions premised on folktales, television series, and documentaries.

## DISCUSSION

There exists a dynamic human–animal interface in the bustling metropolis of Delhi. It is shaped by thousands of avian scavengers such as crows and kites that subsist on waste and food offerings from the residents. This complex interaction, one of the largest of its kind globally, is a testament to the resilience of nature in urban landscapes. It also underscores the importance of monitoring links in the ecological and cultural processes, which have far-reaching consequences in ensuring the health and well-being of both human and non-human animals (Gaston 2010, Soga and Gaston, 2016, 2020). Our thematic analyses (Figure 2), which we discuss in detail below, has allowed us to explore complex human–animal interactions. In our exploration of the diverse cultural mosaic of Delhi’s society, we found that the cultural significance of avian scavengers—and of animals in general—extends far beyond their utilitarian or socio-economic value. Cultures mediated by socio-economic endeavors were evident in the widespread portrayal of common urban birds as shared natural heritage by multiple stakeholder groups in the city. Diverse associations of urban fauna among multiple communities, based on ecosystem services that foster mutualistic relationships among people, nature, and the divine, defined how people shared cities with non-human animals (Cox et al. 2018, Dhee et al. 2019, Gosler and Tilling 2021). However, under the recent global revolutions, the interplay of colonization, modernization, urbanization, and technification, has disrupted vital people–nature connections. These disconnections are particularly stark today, given the extinction of experience associated with the decline in populations of once-common avian species such as vultures (Prakash et al. 2003), sparrows (Nath et al. 2022), and the progressive reduction of bird diversity in urban backyards (Marzluff 2017).

In Delhi, people’s psychological and behavioral responses toward biota were collectively shaped by direct interaction and indirectly through biocultural information, especially via stories. Urbanization increasingly threatens these links, severing connections to nature that can lead to the erosion of cultural identities (Pretty et al. 2009, Soga and Gaston 2016) with respect to how certain communities find specific interactions like bird feeding an expression of their autonomy. Such practices highlight the importance of intangible aspects of eco-literacy, and the understanding of the interdependence of human lives and ecosystem(s) in preserving these connections through cultural notions. In this regard, our study identified three emergent dimensions that are likely driving ongoing changes in folk perceptions. The comprehension of these dimensions can offer valuable insights for relationships between humans and animals within novel (urban) ecosystems. Furthermore, it can serve as a guiding principle in fostering convivial coexistence (Hinchliffe and Whatmore 2006), underscoring the paramount importance of eco-literacy in shaping sustainable interactions with the natural world (Locke et al. 2013).

### The Semantics of Folk Taxonomy

Folk taxonomy is a type of classification that is based on common knowledge and beliefs, remotely based on scientific principles (e.g., habit-habitat linkages described by Dave 2005). Concerning natural history and cultural knowledge, such descriptions suffer from inaccuracy (Atran 1998, Beaudreau et al. 2011, Coley et al. 1999, Lopez et al. 1997). Our ethnographic analysis revealed that ecosystem services, calls and color (or aesthetics), and habit-habitat linkages formed the basis of bird identification/nomenclature across diverse groups, in the decreasing order of importance. These results are consistent with the names and adnouns for birds in Hindi and Sanskrit (see Dave 2005). Kites and other raptors that show nesting site fidelity (Kumar et al. 2018a) may have supported the plausible public notion of a long life (see Newton 1979) for these facultative avian scavengers discussed in *Hindu* scriptures. It earned them the moniker of grandmother of kites/birds that surfaced multiple times in our interactions with the respondents. These observations are consistent with similar popular legends that discuss mammalian commensals like domestic cats and large predators like leopards that often visit urban areas: domestic cats are called “mausi of all large cats,” meaning the maternal aunt of all large cats (Dhee et al. 2019). Regardless of the diversity in their religious beliefs, Indian society is inherently collectivistic, fostering social cohesion and interdependence. This was evident in the unique names respondents used to depict paternal and maternal relations, a feature that distinguishes South Asian populace from Western societies (Chadda and Deb 2013). We propose that these anthropomorphic instances of extending familial relationships to common birds and other fauna (see Aiyadurai 2016, Dave 2005) can aid urban conservation efforts by promoting the concept of universal brotherhood inclusive of the non-humans. Further research is needed to understand how this anthropomorphic rationality for animals contributes to fostering higher tolerance for animals regularly involved in conflicts across South Asia (Pretty et al. 2009; Beaudreau et al. 2011; Medin and Bang 2014; Skandrani et al. 2014).

The confusion in the nomenclature used by people for various raptors, such as referring to kites as eagles or *baaz* (hawks/falcons), underscores the emphasis on the life-history traits of obligate predators. Eagles, symbolically associated with power in regional and global texts (see Dave 2005), are generally not found within human-dominated landscapes. This absence, coupled with the eagles’ renowned hunting prowess (Naoroji 2006), may have led to misidentification. The similarities in plumage and beak shape between eagles and kites, the prominent appearance of eagles, falcons and hawks in television documentaries as charismatic species, while we have limited scientific understanding of tropical urban ecosystems that get limited screen time, may further contribute to this confusion. This phenomenon illustrates how names can reflect aspects of life history traits and how physical resemblance can influence perception and nomenclature. In the wake of the decline in vulture populations that were linked to landfills and carcass dumping sites, large flocks of migratory *M. m. lineatus* (Black-eared Kite) from the Central Asian Steppes have occupied these foraging territories (Kumar et al. 2020b). Birds associated with carcasses, including vultures, have been collectively perceived as harbingers of misfortune by Hindus, with prevalent beliefs associating a perched vulture with impending devastation or death (see Dave 2005). Conversely, vultures also played a crucial role in the cultural ecosystem services for the Parsi community by facilitating sky burials through their consumption of corpses (Naoroji 2006, Van Dooren 2010). This underscores the need for a nuanced urban conservation strategy that recognizes complex human–animal interactions, and the ecosystem services urban animals provide (see Sumasgutner et al. 2021). Such an approach—that keeps eco-literacy at the core of biodiversity conservation in urban settings—is crucial for conviviality (Hinchliffe and Whatmore 2006, McBride et al. 2013).

While there were positive connotations associated with avian metaphors in Indian society, such as *Cheel si aankh* (vision like a kite) and *kaak cheshta* (perseverance like a crow) (see Dave 2005), the practice of zoomorphism—attributing animal characteristics to humans—reveals insidious forms of discrimination. People in cities like Delhi—as revealed in our interviews—use idioms and phrases inspired by avian scavengers, which can have racial hints that are often overlooked in literature. Outcomes of our ethnographic research has indicated that zoomorphism holds the potential to dehumanize, a phenomenon often associated with unintended or overlooked elements of social oppression. In such instances, individuals associated with waste or refuse are perceived similar to (avian) scavengers. These practices frequently contribute to the perpetuation of casteism and religio-cultural stigma. Such stigma often manifests as a shared disdain for scavengers and those engaged in waste management, drawing a concerning parallel between societal and ecological detritus (Kain 2018). Informal waste disposal practices and the congregation of specific urban subalterns and opportunistic scavengers are subjected to regional “otherness” or “alienation.” The gaps in urban planning regarding solid waste and public health management often leave these responsibilities to informal stakeholders, who consequently face inhumane conditions and alienation from the city (Gidwani 2013). Through this paper, we urge people to embrace these facets of their society, fostering a more inclusive and harmonious urban environment.

Indian society has been praised for its anthropomorphic treatment of animals. People attribute human characteristics to animals and foster religious sentiments (Dhee et al. 2019, Galushin 1971, Kumar et al. 2019c). Anthropomorphizing animals, rivers, mountains, and other entities in Indian society has been extensively documented and widely recognized. But, the inverse process of zoomorphism, especially when it serves as a basis for casteist slurs, has largely been overlooked. The implications of zoomorphism, particularly its role in perpetuating casteism and racial slurs, necessitate further investigation (Mishra 2015).

### Mediatization of Perceptions in SETS

Perceptions of animal species within SETS are often shaped by mythological and anthropomorphized perspectives depicted and popularized by media, sometimes even superseding perceptions formed by actual encounters, like the situation in Delhi (Fukano et al. 2020, Fukano and Soga 2021, Soga et al. 2020). The emergence of televised media like National Geographic or Discovery documnetaries, and media series that delve into fiction and mythology such as the Jungle Book, *Ramayan*a, *Arabian nights*, and *Mahabharata*, as well as documentaries featuring religious figures like *Sikh* guru *Gobind Singh ji*, who is often portrayed with an *Accipitrid* raptor (Singh 2021), has enabled the visual representation of characters that were previously confined to the realm of imagination (Rajagopal 2009). The legend of the *Sikh* guru and his pet raptor, colloquially referred to as *baaz*, has influenced modern depictions and public displays of religious clerics. The common perception, as articulated by our interviewees, is that *baaz* is a bird of prey “that exclusively hunts for its own sustenance, often capturing its quarry in mid-air” (Singh 2021). This image, however, is frequently conflated with the sightings of juvenile *Govinda,* or *Lineatus* kites. The streaked breast feathers of these species bear a striking resemblance to those of falcons and hawks. Further, this misidentification might also be influenced by the observed behavior of these kites in urban environments. As documented by Kumar et al. (2014, 2018a), kites have been observed catching ritually tossed meat in mid-air—and hunting pigeons and other pests in the city, often capturing them mid-air—behaviors that closely mirror the hunting style of *baaz*. This phenomenon underscores the role that media plays in shaping cultural perceptions and interpretations of avian species.

While the influence of mythological animal forms on perceptions has been somewhat overlooked by researchers studying SETS, these pathways of perception differ from the perspectives based on direct encounters, popular scientific notions, and systematic identifications practiced in the West (Fukano et al. 2020, Fukano and Soga 2021, Soga et al. 2020). This homogenization of perceptions is a product of fictitious opinions circulated through the internet, visual, and print media (Barua 2010, Thaler and Shiffman 2015). For example, *Panchtantra* and *Aesop*’s Fables, often presented as illustrated stories for children, have contributed to the belief that *C. splendens* possess superior cognition, with 83% of interviewees (*n* = 826) basing their notions on these tales (Dave 2005, Fukano et al. 2020, Fukano and Soga 2021, Soga et al. 2020). Folk stories emerging from social media influenced the beliefs of our respondents regarding bird migration and the decline in vultures. In the absence of adequate advocacy to prevent the use of diclofenac, urban poor respondents in our surveys—who were livestock owners—associated these declines with technological interventions by “western scientists, who took the birds to the United States of America, China, or Japan.” They cited local newspapers and social media as information sources (Blokhin and Ilchenko 2015, Udeze and Uzuegbunam 2013).

The pervasive influence of stories and fables, often favored for their ease of verbal explanation or artwork, can lead to a homogenization of perceptions about urban animals. This underscores the need for a nuanced understanding of the role of the media in shaping perceptions and attitudes toward urban wildlife. However, the potential of mediatization to reinforce patronizing perceptions can contribute to preserving the socio-cultural and ecological uniqueness of tropical megacities, akin to the role of urban green spaces (Kumar et al. 2018a). In this context, the integration of natural history observations with media depictions emerges as a compelling avenue for conservation efforts. Such an approach can effectively link these aspects, providing a more comprehensive and accurate representation of urban wildlife. In regions where religious motivations already inspire the sharing of spaces to conserve biodiversity, scientific discourse alone may not sufficiently stimulate public will. Strategic uses of media can potentially harness these religious sentiments to support urban biodiversity conservation (Nolan et al. 2022).

### Inductive Opinions: The Role of Folk Stories and Social–Ecological Experiences

Perceptions and narratives played a significant role in identifying (intangible) social environmental discontinuities (Angeler et al. 2016), and understanding social–ecological experiences, particularly in rapidly urbanizing systems such as Delhi (Atran 1998, Lopez et al. 1997, Raven et al. 1971). Our interviewees expressed *biophilia* through feeding and anthropomorphism, influenced by stories passed down within societies through generations that served as the primary driver of people–nature connections (see Table 1; Anderson 2001, Bang et al. 2007, Medin and Atran 2004). We suggest that the richness of eco-cultural narratives, which formed an integral part of the biocultural heritage for each species under study, could serve as a gauge for their synurbic status (Hulme-Beaman et al. 2016), thereby mirroring the extent of their anthropophily (Table 1). For example, *C. splendens* that thrives in human-dominated environments, demonstrate a remarkable adaptability in establishing their habitats close to human settlements and in utilizing resources created by human activities. This adaptability is mirrored in the multitude of vernacular names used by our respondents and the fables that feature them (Dave 2005). On the other hand, species like kites and vultures display a comparatively lower degree of behavioral flexibility, especially in their choice of nesting substrate (Naoroji 2006). In their study, Kumar et al. (2018a) discovered that kites never chose to nest on buildings, with only a small percentage of nests found on man-made structures. Conversely, crows extensively utilized artificial structures for nesting, showcasing their opportunistic nature, which is further facilitated by their relatively pronounced omnivory. Given that people associated crows as their ancestors’ reincarnation in Delhi and in most parts of India, perceptions may indicate a species’ behavioral plasticity, synurbic status and adaptability to human proximity. Together they influence its eco-cultural salience within human societies (Kumar et al. 2019c).

Fiction serves as a medium to abstract and simulate social-ecological experiences, reflecting the frequency of interaction events (Mar et al. 2006). Interestingly, even in the absence of direct experiences of harm caused by kites, these birds were widely demonized as “eye-snatchers” by our interviewees. Similarly, crow’s exceptional cognitive abilities are universally recognized, even without firsthand observations of these birds replicating the act of drinking from a partially filled pitcher, a feat depicted in *Aesop’s* (Greek) and *Panchatantra’s* (Indian) fables featuring animals as protagonists (Dave 2005). These instances underscore the patterns of inductive inference. By inductive reasoning, we refer to the cognitive process where individuals extrapolate generalizations from a specific instance or category to a broader one (Coley et al. 1999). This uncanny human propensity for inductive reasoning often finds expression in eco-cultural stories, thereby popularizing certain perceptions about specific species like the “smart” crow.

Urban ecosystems, with their diverse infrastructure, green spaces, and waste dispersion patterns, offer a range of opportunities for stakeholders to interact with urban wildlife. However, these interactions are not evenly distributed. For example, informal waste segregators on landfills, a key stakeholder group in this study, regularly work close to crows, kites, and other animals (Kumar et al. 2019c). While the responses of male waste workers did not differ from those of other urban poor stakeholders, female waste workers had limited opinions or perceptions to describe over 10,000 kites that gathered around landfills during winter (Kumar et al. 2020b, Naoroji 2006). This disparity underscores the gendered nature of interactions with urban wildlife and highlights why our urban conservation strategies need to be furthermore inclusive. For instance, Bang et al. (2007) and Keltner and Haidt (2003) have proposed fostering social cohesion by popularizing native emotions about nature. Encouraging community-driven cultural expressions that celebrate nature can help separate emotions from problems, thereby promoting psychological well-being. Since respondents across the socio-economic classes expressed their concerns for the non-humans and acted in multiple ways, patronizing avifauna, the importance of such biocultural expressions that cater to the well-being of non-humans may help the urban poor immigrants. Scientists, therefore, should be encouraged to collaborate and explore innovative ways in which a changing society rejuvenates its emotions for the non-human life world. Such innovations would help in preserving traditional ecological knowledge within urban immigrants (Manfredo and Manfredo 2008).

## CONCLUSION

### Implications for the Future of Human–Animal Coexistence in South Asia

Ultimately, the scientific (western) concept of species does not represent “real objective units” (Darwin 1859, Mayr 1996) but humanistic constructs of varying accuracy. In the same manner that taxonomists utilize an array of morphological and other indicators to categorize and denominate a species, popular (folk) perceptions and practices are the culmination of diverse viewpoints that people develop through prolonged coexistence (Kumar et al. 2019b). These popular (folk) perceptions and distinct identities associated with commensals emphasize the necessity of integrating human socio-cultural dimensions to augment ecological knowledge and bridge the gaps between various disciplines in the context of urban sustainability (Barua 2014a, b).

In western temperate cities, socio-cultural agglomeration and heterogeneity are less pronounced when compared to tropical regions that house most of the urban impoverished population (Ghosal and Kjosavik 2015). Considering the presence of ritually motivated people throughout all stakeholder groups, comprehending folk dynamics and patterns of inductive generalization assumes heightened significance (Kumar et al. 2019a, 2019c). Furthermore, there is a pressing necessity to document intangible heritage about human expressions to nature held by urban immigrants who have been displaced from their native lands. As nuclear urban societies proliferate, the value of contexts in which the elderly, who frequently lack the suitable means to transfer their knowledge, is becoming increasingly recognized (Kumar et al. 2018a, 2020a, Tidemann and Gosler 2012). The incorporation of social scientific methodologies with ecological viewpoints will contribute to the enhancement of the concept of biological carrying capacity by incorporating factors that influence human ecology (Walker 2006).

As a result of the rapid pace of development in South Asia, the majority of the region’s avifauna either resides within human-dominated landscapes or has habitats that are surrounded by them. The interplay of socio-cultural practices, the presence of numerous small and disjunct protected areas, and the opportunistic response of wildlife to anthropogenic resources necessitates the adoption of a more pertinent social carrying capacity for the human-use landscape (Ghosal and Kjosavik 2015). The pervasive influence of cultural and ritualistic elements in societal dynamics not only fosters tolerance for creatures engaging in confrontations within urban environments but also extends this phenomenon to urban-peri-urban (Wilson et al. 2013; Barua 2014a; Aiyadurai 2016; Dhee et al. 2019) and village-protected area ecotones (Ghosal and Kjosavik 2015). Considering the tendency of contemporary systematic biology to promote the expansion and generalization of scientific research findings, the integration of traditional identities, perceptions, and practices that foster coexistence could enhance conservation initiatives (Nazarea 2006). Such approaches could transcend political boundaries in an era where public will is essential (Kumar et al. 2019c). The significance of this notion is particularly pronounced in the Global South, where traditional methods of identifying strategies to adapt and share living spaces with non-human life forms play a pivotal role in shaping societal perceptions of these life forms (Kumar 2013). This holds particular relevance in light of the nascent stage of (scientific) ecological research on the human–animal interface and its transformation due to rapid urbanization (Marzluff 2017).

Within the socio-dynamic parameters of urban informal settlements, biocultural conservation requires acknowledgment of the urban poor’s deprivation of autonomy and cultural expressions in relation to nature. The prevalence of ritualistic animal feeding not only serves as a means of subsistence for the urban poor in Delhi and South Asia but also highlights the significance of these practices in shaping human–animal interactions (Taneja 2015). Despite recent advancements in eco-literacy approaches that incorporate urban biodiversity, integrating ecological and human science perspectives remains an ongoing endeavor. Our research findings, corroborated by established literature, unveil significant lacunae in interdisciplinary eco-literacy discourse among experts representing various domains such as psychology, anthropology, education, and conventional biological disciplines. Consequently, incorporating the ecosystem concept into the context of tropical megacities necessitates a comprehensive understanding of the spatial dimensions of social differentiation, analogous to patch dynamics in ecology (Pickett et al. 2001).

Finally, the urban poor and nature have frequently encountered limited spaces and facets for complete self-expression amidst rapid transformations UNESCO (2016). This emphasizes the imperative for more inclusive, accessible, and just approaches to urban conservation and sustainability. Narratives and stories actively shape perceptions and attitudes and have played a pivotal role in human evolution by fostering group coordination and cooperation (Smith et al. 2017). Hence, acknowledging the adaptability of storytelling ought to also foster collaboration among various stakeholders to achieve conservation and sustainability objectives. In addition to the preservation of folklores, we propose the dissemination of scientific studies as folk narratives through various media, thereby rendering them accessible to laypeople. This approach could promote biophilia, cooperation in biodiversity conservation, biocultural revitalization, and conflict mitigation.

## Supplementary material

Supplementary material is available at *Ornithological Applications* online.

duae056_suppl_Supplementary_Material

## Data Availability

The dataset is available in supplementary material.
