# Supplementary material for: Feathers, folklore, and eco-literacy: Stories ascribe cultural keystone status to avian scavengers in South Asian cities
Source: Ornithol Appl. 2024 Oct 15;127(1):duae056. doi: 10.1093/ornithapp/duae056 (PMC11822674; doi:10.1093/ornithapp/duae056)
Supplement: duae056_suppl_Supplementary_Material [file duae056_suppl_supplementary_material.docx]

**Supplementary material:** S1, S2 and S3

**Feathers, Folklore, and Eco-Literacy:**

Stories ascribe cultural keystone status to avian scavengers in South Asian cities

**Fig. S1**. Research team explaining scientific objectives and informing people of Delhi on the ecological importance of predatory kites (as well as other birds) while ringing kite chicks. This sort of people assembles constantly in a megacity of more than 30 million inhabitants like Delhi, as we move from nest to nest during our routine fieldwork activities, offering a unique opportunity to reach thousands of people in disseminating science, and educating the population on urban environmental issues of conservation importance. Since 2012, we have reached more than 54,000 citizens through this mobile conservation education initiative, which we see as a mobile laboratory. For many of these people, this may be one of their few opportunities to empathize and get in contact with nature. Still, our conversations with people have often surprised us by the depth of concern they already have, for example, the decline in numbers of small birds that they have noticed over recent years. We, therefore, conducted a structured ethnographic study to document and understand their knowledge and how it integrates into their belief systems.


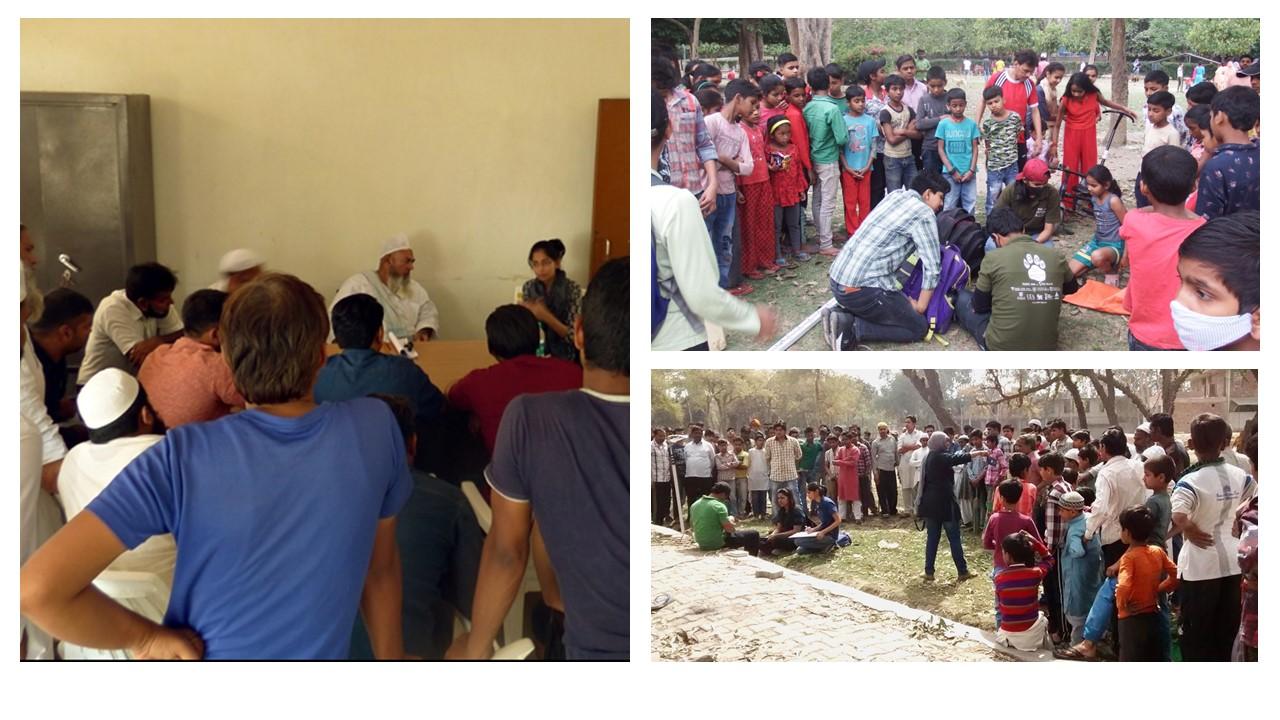


**S2: Description of stakeholder choice while identifying 71 interviewees, and semi-structured questionnaire for this study in the city of Delhi (India).**

| **Stakeholder Groups (number of respondents)** | **Description, rationale for choice and predicted trends in levels and types of association** |
| --- | --- |
| **Butchers** (n=3) | They belong to the informal labor sector and are confined to slaughterhouses while working. This allows them to have significantly close interaction with prominent opportunistic scavengers daily. Additionally, in Delhi, they also feed the choicest portions of meat to kites and other animals to request relief from the sinful act of slaughter. Most of my respondents were migrants from neighboring states |
| **Rag-pickers/slum-dwellers (n=6)** | They have been noticed working at garbage dumps based within residential blocks and on landfill sites. Rag-pickers were expected to have regular close encounters with scavenging birds. It was also very likely that these people had recently immigrated to the city and had a very different native basis of association with the avian scavengers in the city. |
| **Civic bodies (Governmental) - officials related to urban fauna and waste management – Municipal Corporation of Delhi (MCD) and Public Works Department (PWD) (n=4)** | Respondents from this group are involved in the planning and implementation of garbage maintenance and managing green cover. As major stakeholders, they were chosen to understand the consideration officials have for urban biodiversity; and also, to understand political ecologies in relation to the human-animal conflicts involving the target species. |
| **Bird watchers and Academic experts (n=7)** | This group included individuals who observed birds as a hobby/professionally. This group was also chosen to understand if commonly scavenging target species have their attention. |
| **Non-governmental organisations (NGOs) and animal welfare groups:** Jain Bird Hospital, Wildlife SOS, World Wildlife Fund for Nature (WWF, India), Wildlife Trust of India (WTI) (n=10) | Respondents from this group are professionally associated with animal welfare and conservation sectors. I targeted them to understand how much attention large conservation organizations extend to common urban species. It also allowed me to understand the individual opinions of regional experts at the helm of science and policy of conservation |
| **Tourists and employees at Delhi Zoo (n=20)** | Delhi Zoo is a centre of environment and conservation education. Visitors from within and outside Delhi come to the zoo as (I) a centre of learning and sharing with children and (II) a tourist attraction. This group was vital under the expectation that such visitors would be inclined to discuss their appreciation for animals while zoo employees have a regular commitment towards animal welfare and conservation education. |
| **Resident citizens (n=20)** | People who were born in Delhi treat themselves as long-term permanent residents of the capital and not as immigrants from elsewhere. This group allowed us to understand the perceptions of respondents as residents whose native folk biological expressions to animals were assumed to be urban. |
| **Priests/ employees of temples and mosques (n=4)** | Representatives of their religion/culture, this group allowed us to understand and verify socio-religious perceptions other people had about target species, and to factor differences, if any. These stakeholders are vital in orienting the public about ritual feeding to birds and other species, often citing the need as a religious custom suggesting relief from sins, treating them as bad omens, etc. |
| **Respondents of multiple age categories**  **(18-80 years of age)** | We expected the extinction of experience and memory in younger generations as compared to older ones. |
| **General Questionnaire (semi-structured):**   1. Do you feed birds? Which ones and why? 2. Have you seen vultures? What were they like? Were they important? How do you associate with them culturally? 3. What about Black Kites? How do you feel about them? How do you associate with them? 4. And crows? How do you feel about them? How do you associate with them? 5. What do you think about the Population trends of these birds? 6. Are there stories about them that you would like to share?   **Questions asked to birdwatchers/ conservationists, apart from questions posed above**   1. How did you get into birdwatching/ conservation? 2. What is it about birds that interest you? (for these three groups of avian scavengers?) 3. What is the scenario of urban ecology and conservation in India? | |

**S3: Summary of the etymology of folk names given to the target research species, namely Black Kites, Crows and Vultures, and the associated urban legends and major perceptions of citizens within the city of Delhi (India).**

| **Species** | **Morphology** | **Call** | **Names given on color/call** | **Familial structure in the animal kingdom** | **Name given of ecosystem service or extinction of experience** | **Experience** | **Story/urban legend** | **Religious symbol** | **Perception** |
| --- | --- | --- | --- | --- | --- | --- | --- | --- | --- |
| Crow | Black, straight beak | Distinct cawing | Kauva, kaag, kaak, crow |  | Same | Feeding at home, attack during failed nesting attempt | Crow and the pitcher; blind by one eye as Lord Ram shot one in Ramayana, crow’s caw signals guest arrival; crows go to far-off places to die | Messenger of God of death (Yam), Vehicle of Shani, form of deceased ancestors | Mystery, cunning,  intelligence, |
| Kite | Dark coloured, hook-like beak | Shrill whistle | Cheel,  Baaz | Pakshiyon ki naani (grandmother of kites) | Eagle,  Giddh/gradh (vulture) | Snatches food from hand, attacks during nesting season, elegant flight | If you look at them, they will snatch your eyes, feeding them takes away sins | Symbols in Rajputs and Kashmiri pandits | Strong birds, powerful eyesight,  Angel from heaven, bad omen |
| Egyptian vulture | White | - | Safed cheel | Cheelon ki naani | Safed cheel, chhote giddh | Feed on carcass |  |  | Powerful eyesight |
| Other vulture species | Long, bare necks, large wingspans | - | Giddh | Giddh | Giddh | Used to clean a carcass, once present in large numbers, now rarely seen | The population declined due to pollution, technological advancements | Garuda and Jatayu | Powerful eyesight, large distance fliers,  Bad omen |

**Fig**: **Pamphlet Circulated By The Charity Jain Bird Hospital To Support Feeding Of Animals And Its Translation**


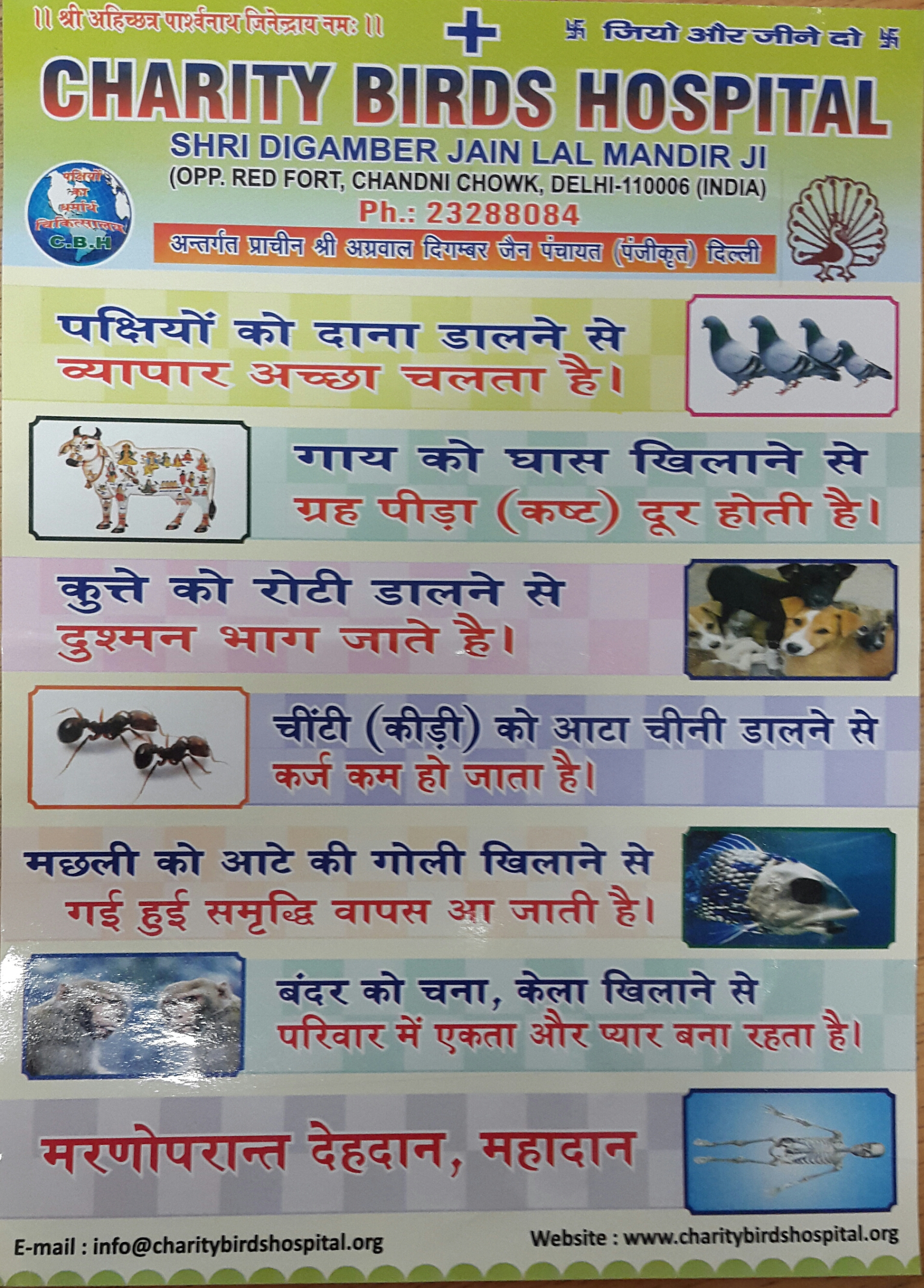


| Feeding grains to birds will benefit employment |
| --- |
| Feeding grass to cows will keep problems away |
| Feeding Chappatti to dogs will keep enemies away |
| Feeding wheat flour and sugar to ants frees you from debt |
| Feeding dough balls to fish returns lost wealth |
| Feeding chickpeas and bananas to monkeys keeps the family strong |
| Organ donation is the biggest donation |
